# Validity, reliability and acceptability of Professionalism Mini-Evaluation Exercise (P-MEX) for emergency medicine residency training

**DOI:** 10.18502/jmehm.v12i12.1641

**Published:** 2019-10-15

**Authors:** Leila Amirhajlou, Ali Bidari, Fateme Alipour, Mehdi Yaseri, Samira Vaziri, Mahdi Rezai, Nader Tavakoli, Davood Farsi, Mohammad Reza Yasinzadeh, Reza Mosaddegh, Akram Hashemi

**Affiliations:** 1 *Researcher, Department of Medical Education, School of Medicine, Tehran University of Medical Sciences, Tehran, Iran. *; 2 *Professor, Department of Emergency Medicine, School of Medicine, Iran University of Medical Sciences, Tehran, Iran. *; 3 *Associate Professor, Department of Ophthalmology, School of Medicine, Tehran University of Medical Sciences, Tehran, Iran.*; 4 *Associate Professor, Department of Biostatistics, School of Public Health, Tehran University of Medical Sciences, Tehran, Iran.*; 5 *Assistant Professor, Department of Emergency Medicine, School of Medicine, Iran University of Medical Sciences, Tehran, Iran. *; 6 *Assistant Professor, Department of Emergency Medicine, School of Medicine, Iran University of Medical Sciences, Tehran, Iran. *; 7 *Associate Professor, Trauma and Injury Research Center, Iran University of Medical Sciences, Tehran, Iran. *; 8 *Associate Professor, Department of Emergency Medicine, School of Medicine, Iran University of Medical Sciences, Tehran, Iran. *; 9 *Assistant Professor, Department of Emergency Medicine, School of Medicine, Iran University of Medical Sciences, Tehran, Iran. *; 10 *Assistant Professor, Department of Emergency Medicine, School of Medicine, Iran University of Medical Sciences, Tehran, Iran. *; 11 *Assistant Professor, Department of Medical Ethics, School of Medicine, Iran University of Medical Sciences, Tehran, Iran. *

**Keywords:** Medical professionalism, Emergency medicine, Residency program, Workplace-based assessment

## Abstract

Professionalism is a core competency in the medical profession. In this paper, we aimed to confirm the validity, reliability and acceptability of the Professionalism Mini-Evaluation Exercise (P-MEX) instrument for the emergency medicine (EM) residency program. Twenty-two EM attending physicians completed 383 P-MEX forms (the Persian version) for 90 EM residents. Construct validity was assessed via structural equation modeling (SEM). The reliability coefficient was estimated by the generalizability theory, and acceptability was assessed using two researcher-made questionnaires to evaluate the perspectives of residents and assessors. There was a consensus among the participants regarding the content of P-MEX. According to the results of SEM, the first implementation of the original model was associated with a moderate fit and high item loadings. The model modified with correlated error variances for two pairs of items showed an appropriate fit. The reliability of P-MEX was 0.81 for 14 occasions. The perception survey indicated high acceptability for P-MEX from the viewpoint of the residents and increasing satisfaction with P-MEX among the assessors over time.

According to the results of the research, P-MEX is a reliable, valid, and acceptable instrument for assessing professionalism in EM residents.

## Introduction

Professionalism is a core characteristic of the medical profession (1). In recent years, increasing attention has been paid to professionalism due to concerns regarding the decline of professional and ethical values (2). One responsibility of medical schools is determining whether such competencies have been achieved (3). Over the last three decades, various instruments have been developed to assess medical professionalism (4). Recognizing the observation of students’ performance as the most efficient technique to evaluate professionalism in real clinical practice led to the identification of the Professionalism Mini-Evaluation Exercise (P-MEX) as the core of any assessment strategy (5). 

Introduced by Cruess et al., P-MEX measures four areas of professionalism skills: doctor-patient relationship, reflective skills, time management, and inter-professional relationship skills (6). The necessity to reevaluate professionalism assessment scales before application in a new country has been emphasized due to cultural and contextual differences (7,8). For instance, Tsugawa modified the instrument so that it could be applied to Japanese medical students (9,10). Unfortunately, no observational instrument has been validated for the assessment of the professionalism of emergency medicine (EM) residents (11,12). Working as a resident in the EM department is more stressful compared to other departments due to the unique features of this ward, e.g. heavy workload, uncontrolled environment, and an unlimited number of patients with a vast spectrum of diseases and a short-term stay (13).

Studies have shown that professional values are violated by residents who suffer from burnout due to prolonged exposure to stress. Formative assessment of behavior facilitates early identification of unprofessional behavior before it becomes a significant issue. It also assists trainers in opening the dialogue on signs of burnout with residents through feedback for minimizing professionalism lapses and ameliorating burnout. Therefore, it is essential to apply effective assessment strategies in the clinical workplace (14,15). Considering the differences between the EM department and other clinical settings, this study aimed to confirm the reliability, validity, and acceptability of the P-MEX for EM residents in Iran.

## Methods

This study was conducted in the EM departments of four teaching hospitals in Iran from July 2017 to January 2018. The research was approved by the Institutional Review Board of the School of Medicine of Tehran University of Medical Sciences. In translating the P-MEX from English to Persian, the guidelines for the translation and adaptation of tests developed by the International Test Commission (ITC) were followed (16). First, two experts conducted a forward translation, which was then evaluated by an expert panel consisting of five professionals. This evaluation led to the formation of a single Persian translation by consensus. Second, the Persian version was back-translated into English by two bilingual native English speakers. Third, Richard Cruess and Sylvia Cruess (the two developers of the P-MEX) discussed the discrepancies in the two backward translations. Based on their recommendations, a final draft of the Persian P-MEX was prepared. Fourth, cognitive debriefing interviews were conducted with a sample of participants, consisting of six assessors and six EM residents, to assess the comprehension and face validity of the translated P-MEX. 

All EM residents (n = 90) and 22 attending physicians voluntarily participated in the study. Non-monetary incentives were used to encourage participation in the research. Participants were first instructed to perform the P-MEX exercise through weekly meetings, in which they received a booklet containing an instruction guide and the P-MEX forms. The P-MEX comprises 20 minutes of observing clinical encounters followed by five minutes of immediate feedback. In the present study, the full 24-item P-MEX scale was used, scored based on a four-point Likert scale with the options of exceeded expectations (score 4), met expectations (score 3), below expectations (score 2), and unacceptable (score 1). The fifth category was entitled “not observed” or “not applicable”. The original P-MEX form (questionnaire) is presented at the end of the paper as an appendix. 

***Analysis***

The structural equation modeling (SEM) was utilized to investigate the construct validity of the P-MEX. The following indices of SEM were applied in the present study to evaluate the model’s goodness-of-fit: comparative fit index (CFI > 0.90 indicative of a good fit), the root-mean-square error of approximation (RMSEA < 0.08 indicating acceptable fit), and Chi-square (χ2 / d.f. ≤ 3 ratio). SEM statistics were also conducted using the STATA/IC (14.2) (StataCorp, College Station, TX, USA). Moreover, the generalizability theory was used to evaluate the reliability of the scores. To this end, the Generalizability coefficient (G) was estimated for a one-facet crossed design, in which resident (R) was the object of measurement and occasions (o) were facets of measurement using the G-STRING IV version 6.3.8 (Bloch & Norman, 2011). Furthermore, the G study was performed followed by the decision study to identify the number of occasions (P-MEX) per resident required to achieve the highest level of reliability. After the completion of the P-MEX assessment process, residents and faculties were asked to complete a questionnaire on their perception of the P-MEX from various aspects, including the feasibility, content, fairness, and educational impact of the assessment. The questionnaire for residents contained 52 items, whereas the scale for assessors encompassed 37 items, both scored based on a five-point Likert scale ranging from “strongly disagree” (1) to “strongly agree” (5). The face and content validity of the questionnaires were confirmed by a group of experts consisting of two medical education faculty members and four emergency medicine specialists who participated in the study as assessors. In order to determine the stability of the questionnaires over time, test-retest was used by Pearson's correlation coefficient. Consequently, the questionnaires were re-administered to 21 residents and 8 assessors two to three weeks later. In the current study, Pearson's correlation coefficient was between 0.726 and 0.943 for the residents’ questionnaire (*P* < 0.01), and between 0.779 and 0.906 for the assessors’ questionnaire (*P* < 0.01), which suggested satisfactory stability. Furthermore, the reliability of the questionnaires was estimated at the Cronbach's alpha of 0.88. Data analysis was performed in SPSS 22.

## Results

In total, 383 P-MEX forms were completed by 22 EM faculties for 90 residents during a seven-month period. The mean number of evaluations per resident was 4.26 (range of 1 - 11). In addition, the range of the P-MEX completed per rater was 1 - 46, with an average of 17.41 (+/- 2.68 SD). Moreover, the mean of the evaluation scores of all residents for overall competency was 3.32 (± 0.04 SD) out of 4. According to the results, the mean observation time equaled 128.3 minutes (median of 120 and range of 10 - 600) and the mean feedback time equaled 13.06 minutes (median of 10 and range of 1 - 35). In the present research, the residents received the lowest scores on items 10 (23.8%), 8 (21.4%), 17 (14.9%), and 13 (12.5%), which pertained to soliciting feedback, warning about the limitations, addressing the gap between knowledge and skills, and maintaining composure in difficult situations, respectively.

In 11% and 8.6% of the assessments, item 23 (using health resources appropriately) and item 22 (maintaining patient confidentiality) were rated as *not-applicable* by the assessors, respectively. These items were reconsidered for their additional value in the assessment of the EM residents in this research. However, item 22 was more applicable in over-an-hour-long observations. It should be noted that the correlation between intra-item subscales was evaluated using the Pearson product-moment correlation coefficient, and results were indicative of a significant and strong correlation between items 2 and 3 (r = 0.774, *P* < 0.005). Moreover, item 5 was highly correlated with items 4, 6, and 7 (r = 0.773, 0.864 and, 0.743, respectively). 

**Figure 1:**
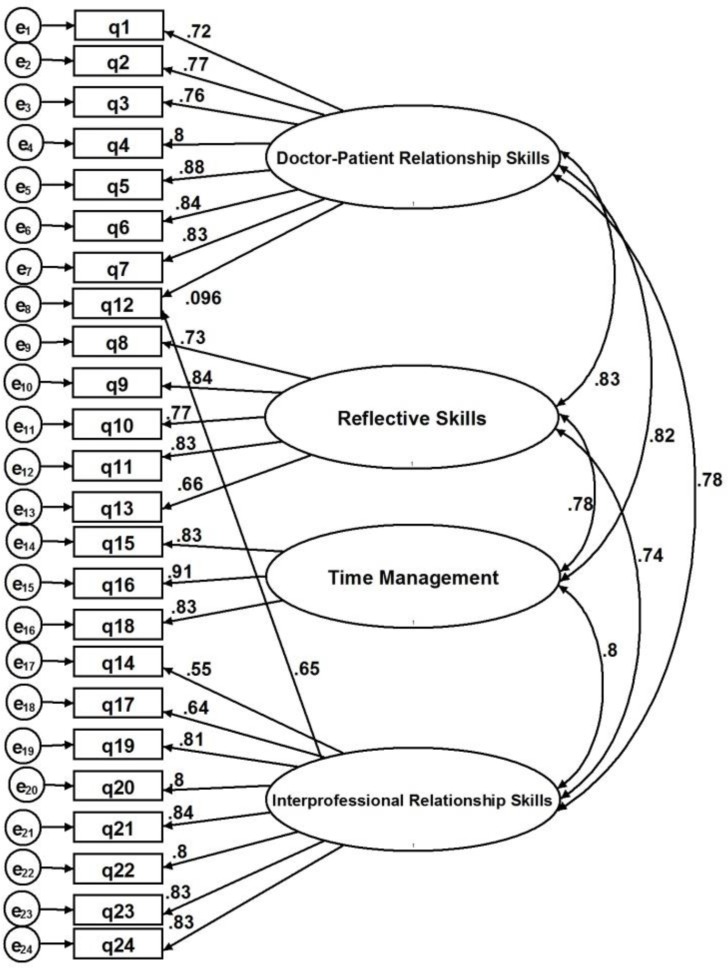
Hypothetical Measurement Model

In addition, SEM was used to confirm the model’s goodness-of-fit. As presented in figure 1, factor loadings for all items were significantly above Kline’s cut-off point (>0.50) (17). However, item 12 (appropriate boundaries with patients/colleagues), had been cross-loaded on two latent variables (i.e. patient-doctor communication skills and interpersonal skills in the original model), and was barely loaded on factor 1 (loading value 0.096) but mostly on factor 4 with a value of 0.65. 

Therefore, based on the results, this item should only be correlated with factor 4 in the modified model. As shown in figure 1, the hypothetical model had a mediocre goodness-of-fit with the following indices: (χ2 = 1124, 964, RMSEA = 0.09, CFI = 0.87, TLI [NNFI] = 0.86). Therefore, we utilized the modification indices given by the STATA/IC software package to provide a model with a better goodness-of-fit. 

**Figure 2:**
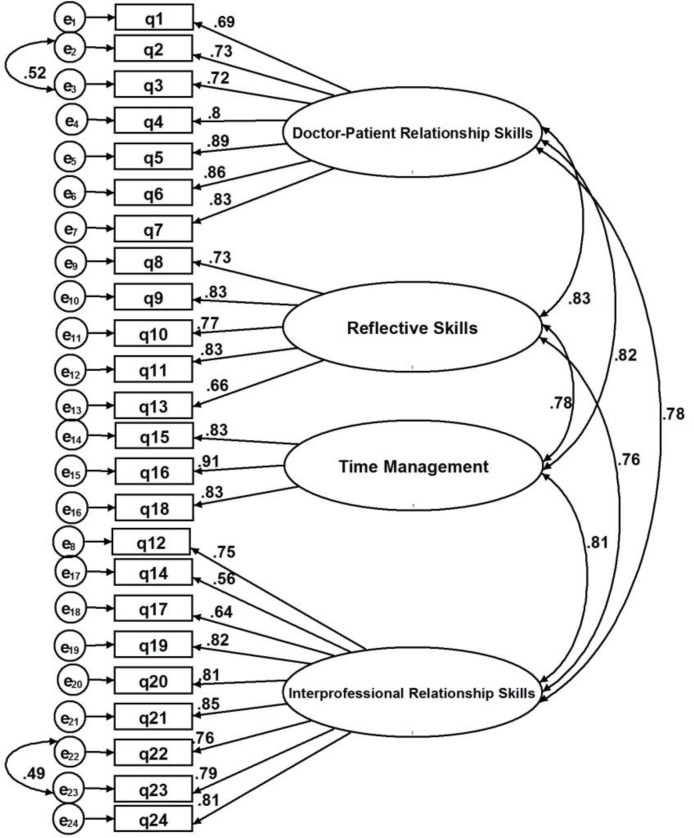
The modified measurement model with correlated error terms, indicating best fit with the study data with χ2 = 955.422, RMSEA=. 0.087, CFI= 0.900, TLI (NNFI)= 0.887.

Modification indices suggested that the value of the model’s goodness-of-fit be elevated by allowing the error term correlations between two sets of items. The error terms of item 2 (showing interest in patient as a person) was correlated with the error term of item 3 (showing respect for patient) by the highest M.I. of 99.431. Moreover, correlations were added across the error terms of items 22 and 23 (maintained patient confidentiality and used health resources appropriately, respectively). The modified model and the correlated errors are illustrated in figure 2, according to which better fit indices were obtained as follows: χ2 = 955.422, RMSEA = 0.087, CFI = 0.900, TLI [NNFI] = 0.887. These indices demonstrated a slight improvement in the model’s goodness-of-fit. 

 Based on the literature, correlated error terms are often caused by item wording, item placement, double-barreled questions, or the effects of missing variables. In order to address the issue of correlated error terms, researchers recommended removing or merging items with the correlated error terms and proposing new items (17- 21). The error correlation between items 2 and 3 could be justified by referring to the same concept of respect for patients with both items. Item 2 was removed from the scale due to the failure of the evaluators to differentiate between the items after scoring the performance of the residents.

Additionally, the error terms of items 22 and 23 were allowed to correlate. However, item 23 did not apply in our setting since it is not the responsibility of residents to use or allocate health resources. As a result, item 23 was eliminated from the scale. The reliability of P-MEX scores was measured by the generalizability theory using a one-facet (the resident by form) crossed design. The G coefficient was estimated at 0.647 based on the six levels of the forms crossed with residents. The D study results (table 1) revealed that the optimal number of occasions required for reaching acceptable reliability on the P-MEX assessment was 14 occasions with the G coefficient equal to 0.81. 

**Table 1:** D Study Results for S×O Design

**Occasions Level**									
	2	4	8	10	12	14	25	28	30
**Ep** ^2^	0.379	0.550	0.710	0.753	0.786	0.810	0.884	0.895	0.902
**ɕ** ^ 2^ **(** **ɕ** **)**	0.57	0.029	0.014	0.011	0.010	0.008	0.005	0.004	0.004

Based on table 2, the acceptability of the P-MEX was measured by a post‐intervention questionnaire. The results indicated that all of the participants were satisfied with the content of the P-MEX. In this regard, 56.6% of the residents responded “strongly agree”, whereas 43.3% selected the option “agree”. On the other hand, 55.6% and 44.4% of the faculties chose “strongly agree” and “agree”, respectively. 

**Table 2:** Faculties’ and Residents’ Perception of P-MEX

	Strongly Agree%		Moderately Agree%		Disagree %		Strongly Disagree%		Undecided %	
**Statements**	F*	R**	F	R	F	R	F	R	F	R
**P-MEX included ** **appropriate content. **	55.6	56.6	44.4	43.4	0	0	0	0	0	0
**Length of the assessment ** **time was appropriate.**	38.9	39.6	38.9	34	16.7	15.1	5.6	5.7	5.6	5.7
**Length of the feedback ** **time was appropriate.**	44.4	32.1	27.8	15.1	22.2	28.3	0	11.3	5.6	2.13
**Assessment was done ** **easily.**	22.2	50.9	61.1	26.4	11.1	17	5.6	1.9	0	3.8
**The busy EM had a ** **negative effect on the ** **assessments.**	33.3	32.1	27.8	22.6	27.8	26.4	11.1	18.9	0	
**Did you have enough time ** **for the assessment?**	38.9	39.6	44.4	41.5	5.6	3.8	11.1	13.2	0	1.9
**Assessment had an adverse ** **effect on the patient care ** **process.**	0	9.1	16.7	13.2	44.4	58.5	27.8	24.5	11.1	1.9
**The feedbacks were useful.**	-	30.2	-	32.1	-	17	-	11.3	-	9.4
**Previous raters' perception ** **of residents had an impact ** **on the** **rating scores.**	38.9	41.5	0	32.1	33.3	17	27.8	7.5	0	1.9
**The quality of the ** **relationship between raters ** **and residents affected the ** **rating scores.**	-	50.9	-	28.3	-	15.1	-	3.8	-	1.9
**The raters were fair on ** **scoring the performance.**	-	11.3	-	39.6	-	28.3	-	1.9	-	18.9
**Do you prefer student-** **centered approach for the ** **assessment process?**	22.2	26.4	33.3	24.5	5.6	24.5	33.3	24.5	5.6	0
**The rater should not make ** **the residents aware that ** **they are being observed, ** **and should use the indirect ** **observation approach.**	50	49.1	27.8	18.9	16.7	15.1	5.6	13.2	0	3.8
**Identity of raters should be ** **unknown to residents.**	50	3.8	16.7	3.8	22.2	35.8	11.1	54.7	0	1.9

*EM= emergency ward; *

* *= Faculties;*

** *=Residents*

As presented in this table, the majority of the participants “agreed” and “strongly agreed” that the P-MEX was easily administrated in EM clinical settings, and confirmed adequacy of the time allocated for completing the questionnaire. Nevertheless, it seems that in some cases, the assessment process was negatively affected by the heavy workload in overcrowded EM settings. In addition, it was found that feedbacks were recorded in only 12% of the P-MEX forms. Moreover, a small number of residents reported only receiving general verbal comments on their performance. Most of the residents and some of the faculties believed that raters’ prior knowledge about resident’s performance creates a positive or negative halo, influencing the grading of the latter’s professional behavior. They also mentioned the effect of the quality of the relationship with raters on the rating scores. Furthermore, more than half of the faculty members preferred the indirect observation of professional behavior in which residents are unaware that they are being observed; most of the residents, however, selected the options “disagree” and “strongly disagree” regarding this statement.

## Discussion

To the best of our knowledge, this was the first psychometric study of the P-MEX in the EM clinical setting. In total, two items were identified as “unfitting and problematic” in the divergent validity analysis. Items 2 and 23 were removed due to the error correlation observed for similar wording and non-applicability. Moreover, item 12 was only loaded on the interpersonal factor. Finally, data were fitted to the proposed model by removing two items. According to the previous studies by Cruess (6) and Tsugawa (9,10), three double-barreled questions (3,7,12), which were confusing and caused bias, had to be divided into six items (6, 9, 10). In the final edition of P-MEX, we corrected these three double-barreled questions.

In the present study, when assessments were performed during the day when patient flow was lowest, time was not a significant issue for either the faculties or the residents. However, they believed that situations with clinical overload or high stress conflicted with the implementation of the P-MEX, since assessment had an adverse effect on the patient care process in life-threatening situations.

Providing feedback was the most significant factor faced by assessors in implementing P-MEX in the present study. While feedback is an essential component of this formative assessment, provision of feedback on the observed clinical performance was inadequate. Since professionalism is subjective in nature, different assessors judge behaviors in different ways and may give different feedbacks, so residents are likely to view a low score and constructive feedback as unfair. In interviews, assessors expressed their interest in providing feedback but had concerns about the emotional and defensive reactions of residents to criticism, which could lead to poor performance in clinical settings. Moreover, they believed that it could potentially cause tension in the supervisory relationship. The working relationship between faculties and residents over an extended period caused leniency bias in ratings in this face-to-face assessment. To address the dilemma existing between the necessity of providing feedback and preventing tension in the busy and stressful emergency setting, assessors suggested the anonymity of raters whereby residents would be aware of the scores and feedbacks but the identity of raters would remain confidential. 

Nevertheless, the residents stated that they were enthusiastic about having an opportunity to learn from feedback and even criticism because it made them understand expectations and identify their own weak points. These findings are consistent with those of Colletti et al. (22) who found that while medical students desire more timely (22), direct observation and feedback on their clinical performance, faculties are unwilling to point out students’ weaknesses face-to-face, particularly when it involves negative feedback, resulting in score inflation. Therefore, there is an obvious need for residents to improve their feedback solicitation skills, and for faculty members to develop their observation and feedback skills with an emphasis on creating a feedback-friendly environment and professional support with mutual trust. 

Another phenomenon observed in the present study was that the mean time of observation was about one hour and a half. Although all raters were instructed on the principles of this assessment, a few were still unfamiliar with the P-MEX instrument and its ultimate goal that is formative coaching rather than assessing, and tended to implement it using the classical global rating methods. They applied the instrument for assessing residents in one shift using the multiple mini-observations technique for the completion of each P-MEX, so that their recorded observation time was about five to ten hours. This affected the total observation time and led to an increase in the average time. They thought that observation should take as long as possible until all items are observed and scored. According to them, some items on P-MEX such as respect for hospital rules, maintaining composure in difficult situations, and ensured continuity of patient care needed an observation time of more than 20 minutes to accurately detect and rate performance. 

Also, the assessors preferred an indirect approach in which residents are unaware of observation, which was inconsistent with the opinion of the residents. Assessors believed that the residents altered their behavior due to the observer's presence, thus undermining the validity of scores. Even though direct observation instruments promise to assess real behavior in workplace situations, observer effects, referred to as Hawthorn or Reactivity Effect, will somehow create bias and make it impossible for raters to rate and document the natural behavior of residents. This is consistent with the findings of Watling et al., exploring the influence of professional culture on the use of direct observation within medical training (23). 

Raters and residents also mentioned that assessors were affected by the halo effect in performance ratings. This means that they applied their general impressions rather than objective ratings of specific behaviors when residents were well known to them. This result is in contrast with the findings of Lie et al., showing accuracy of halo effect with no overall tendency to overestimate the skills of medical students (24). 

Furthermore, residents mostly agreed with the statement that the quality of the relationship with raters affects their scores. All the issues, including lack of feedback, residents’ inflated view of their own professionalism, poor reflective skills, halo effect, and the effect of the relationship with raters influenced the residents’ views of the fairness of the assessment. This led to a distrustful relationship between residents and faculties, so that most of the residents preferred the student-centered approach in which they had the opportunity to choose lenient and safe raters for increasing their scores. 

A review of feedback from trainers demonstrated that the expectations of the attending physicians from residents are beyond the observable behaviors presented on the P-MEX. Some items highly expected of residents were: showing self-confidence; not being either over- or under-confident (grandiosity or low self-esteem, respectively); not being irritable, defensive, depressed, stressed or anxious; being self-motivated; having a sense of responsibility; and actively soliciting and responding to feedback by making action plans for improvement. 

**Figure 3:**
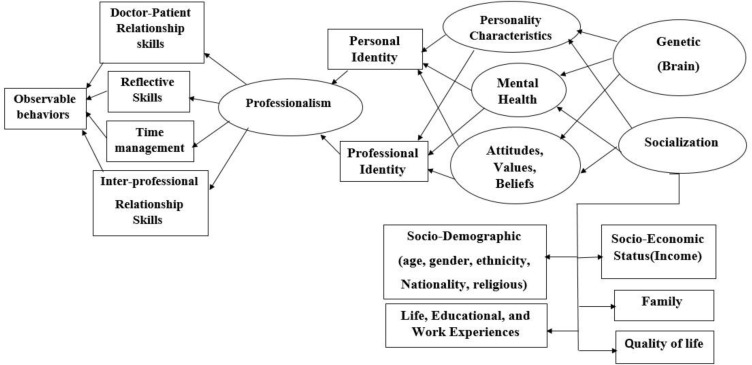
Factorial Model for Assessing Professionalism in Future Studies

These expectations were associated with mental health and stability. Studies have shown that there is a correlation between positive mental health and professional behaviors among medical students. On the other hand, students suffering from burnout are more likely to have engaged in unprofessional behaviors, which subsequently leads to inadequate development of personal and professional identity (25, 26). Cruses et al. stated that the ultimate goal of any professionalism teaching and assessment activity should be personal and professional identity formation (27, 28). Personal identity allows a person to have a sense of uniqueness through a complex combination of socio-demographic and personality characteristics, values, and beliefs (28). The biological perspective on personality confirms that individual characteristics can partially be traced back to the brain and genetic factors; however, through the socialization process, people gain values and beliefs from the society and life experiences, and thus professional identity is shaped (29-33). Therefore, relying on professionalism assessment using only one instrument like P-MEX, which merely measures observable behaviors, may reduce attention to other underlying factors affecting professionalism, and subsequently its final goal, that is, personal and professional identity formation. We propose a more extended model of professionalism measurement in future studies in order to assess the effect of other latent factors (figure 3).

## Conclusion

According to the results of the present study, the P-MEX is a valid instrument on the condition that several modifications are made, including removal and addition of some items. Moreover, the reliability and feasibility of the instrument were confirmed in EM settings. While the P-MEX was highly accepted by residents, faculties were not initially comfortable with the instrument. It became progressively easier as the assessors observed that residents were showing more interest in receiving and soliciting feedback. To accurately assess professionalism among residents, we need to go beyond traditional methods. If consensus is achieved on the fact that the importance of any professionalism assessment lies in professional identity formation, educational goals should be modified so that challenges in the emergency ward, such as heavy work-load and stress, become educational opportunities for residents, resulting in the development of their professional identity rather than burnout and professional insufficiency. It is believed that developing the faculty’s perception of this issue with an emphasis on enhancing their knowledge and skills regarding principles of effective feedback and assessment methods in clinical settings can play an important role in the improvement of the feasibility, acceptability, and validity of the P-MEX.

**Appendix**

**The Original P-MEX Questionnaire**

**Table:** 

Question	Exceeded Expectations	Met Expectations	Below Expectations	Unacceptable	N/A or N/O
Factor 1: Doctor–Patient Relationship Skills	□	□	□	□	□
1. Listened actively to patient.	□	□	□	□	□
2. Showed interest in patient as a person.	□	□	□	□	□
3. Showed respect for patient	□	□	□	□	□
4. Recognized and met patient needs.	□	□	□	□	□
5. Accepted inconvenience to meet patient needs.	□	□	□	□	□
6. Ensured continuity of patient care.	□	□	□	□	□
7. Advocated on behalf of a patient and/or family member.	□	□	□	□	□
12. Maintained appropriate boundaries with patients/colleagues.	□	□	□	□	□
Factor 2: Reflective Skills	□	□	□	□	□
8. Demonstrated awareness of limitations.	□	□	□	□	□
9. Admitted errors/omissions.	□	□	□	□	□
10. Solicited feedback.	□	□	□	□	□
11. Accepted feedback.	□	□	□	□	□
13. Maintained composure in a difficult situation.	□	□	□	□	□
Factor 3: Time Management	□	□	□	□	□
15. Was on time.	□	□	□	□	□
16. Completed tasks in a reliable fashion.	□	□	□	□	□
18. Was available to patients or colleagues.	□	□	□	□	□
Factor 4: Interprofessional Relationship Skills	□	□	□	□	□
12. Maintained appropriate boundaries with patients/colleagues.	□	□	□	□	□
14. Maintained appropriate appearance.	□	□	□	□	□
17. Addressed own gaps in knowledge and skills.	□	□	□	□	□
19. Demonstrated respect for colleagues.	□	□	□	□	□
20. Avoided derogatory language.	□	□	□	□	□
21. Assisted a colleague as needed.	□	□	□	□	□
22. Maintained patient confidentiality.	□	□	□	□	□
23. Used health resources appropriately.	□	□	□	□	□
24. Respected rules and procedures of the system.	□	□	□	□	□

## Notes:

**Citation to this article:**

Amirhajlou L, Bidari A, Alipour F, Yaseri M, Vaziri S, Rezai M, Tavakoli N, Farsi D, Yasinzadeh MR, Mosaddegh R, Hashemi A. Validity, reliability and acceptability of Professionalism Mini-Evaluation Exercise (P-MEX) for emergency medicine residency training. J Med Ethics Hist. 2019; 12: 12.
